# Percutaneous stent placement for malignant hilar biliary obstruction: side-by-side versus stent-in-stent technique

**DOI:** 10.1186/s12876-020-01316-w

**Published:** 2020-06-05

**Authors:** Wei-Zhong Zhou, Sheng Liu, Zheng-Qiang Yang, Yu-Tao Xian, Hong-dou Xu, Jun-zheng Wu, Hai-Bin Shi

**Affiliations:** 1 Department of Interventional Radiology, The First Affiliated Hospital of Nanjing Medical University, 300 Guangzhou Road, Gulou District, Nanjing, 210029 China; 2 Department of Interventional Radiology, Cancer Hospital Chinese Academy of Medical Sciences; Chaoyang District, Beijing, 100021 China; 3 Department of Interventional Radiology, Ningbo First Hospital, 59 Liuting Street, Ningbo, 315010 China

**Keywords:** Hilar biliary obstruction, Bilateral stenting, Jaundice, Stent patency

## Abstract

**Background:**

Currently, side-by-side (SBS) and stent-in-stent (SIS) are the two main techniques for stent deployment to treat hilar biliary obstructions. Previous studies comparing these two techniques are very limited, and thus, no consensus has been reached on which technique is better. The purpose of this study is to compare the clinical efficacy and safety of SBS and SIS deployment via a percutaneous approach for malignant hilar biliary obstruction.

**Methods:**

From July 2012 to April 2019, 65 patients with malignant hilar biliary obstruction who underwent bilateral stenting using either the SBS or SIS techniques were included in this study. Among them, 27 patients underwent SIS stent insertion (SIS group), and the remaining 38 patients underwent SBS stent insertion (SBS group). Technical success, improvement of jaundice, complications, duration of stent patency, and overall survival were evaluated.

**Results:**

Technical success was achieved in all patients in the two groups. The serum bilirubin level decreased more rapidly 1 week after the procedures in the SBS group than in the SIS group (*P* = 0.02). Although the total complication rate did not differ between the two groups, cholangitis was found to be more frequent in the SIS group (*P* = 0.04). The median stent patency was significantly longer in the SBS group (149 days) than in the SIS group (75 days; P = 0.02). The median overall survival did not significantly differ between the two groups (SBS vs. SIS, 155 days vs. 143 days; *P* > 0.05).

**Conclusions:**

Percutaneous transhepatic bilateral stenting using either the SBS or SIS technique is safe and effective in the management of malignant hilar biliary obstruction. However, SBS offers a quicker improvement of jaundice, a lower incidence of cholangitis after the procedure, and a longer stent patency period than SIS.

## Background

Self-expandable metal stent placement is a well-accepted palliative therapy for the treatment of malignant obstructive jaundice [1, 2]. Compared with external catheter drainage, stent placement not only improves patient quality of life but also avoids bile loss. However, stent placement for hilar biliary obstruction is more complicated than that for distal biliary obstruction, as bilateral stenting with two or more stents is often required. Side-by-side (SBS) and stent-in-stent (SIS) are the two most commonly used techniques for bilateral stent deployment [3, 4].

Comparisons of these two techniques are very limited in the literature and the results from previous studies are conflicting [5–8]. There is no conclusive answer to the question of which technique is better. Moreover, all stent deployment procedures in the limited previous studies were performed endoscopically. Thus, it is worth further investigating these two techniques with the percutaneous approach.

The purpose of this study was to compare the clinical efficacy and safety of side-by-side and stent-in-stent deployment via bilateral percutaneous routes for malignant hilar biliary obstruction.

## Methods

### Patient selection and stents

The study was approved by our institutional review board. We retrospectively reviewed the medical records and images of 69 patients who underwent bilateral metallic stent placement using SBS or SIS techniques for the treatment of unresectable malignant hilar biliary obstructions between July 2012 and April 2019 in our department. The inclusion criteria of this study were as follows: 1. diagnosis of malignant unresectable biliary hilar obstruction based on laboratory and imaging or pathological findings; 2. no previous biliary drainage prior to admission; and 3. regular follow up. Four patients were excluded, including three who were lost to follow up and one who received external drainage in another hospital. Of the remaining 65 patients who met the inclusion criteria, 38 patients underwent stent placement using the SBS technique (SBS group) and the other 27 patients underwent stent implantation with the SIS technique (SIS group).

Two types of uncovered self-expandable metallic stents (E-Luminexx (Bard Peripheral Vascular, Tempe, AZ), Microtech (Microtech, Nanjing, China)) with diameters of 8 mm and lengths of 60 mm–100 mm were used in this study.

### Procedure

Prior to the procedure, the biliary hilar strictures were assessed on contrast-enhanced computed tomography (CT) and/or magnetic resonance cholangiopancreatography (MRCP). To relieve pain during the procedure, intravenous sedation with oxycodone was applied.

### SBS Group

**Fig. 1:**
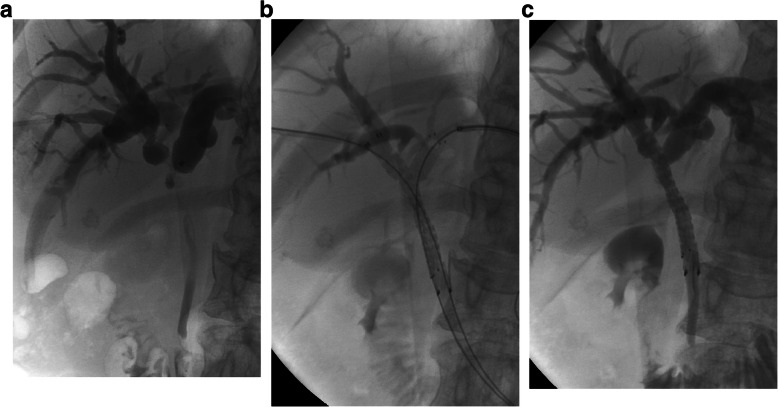
A 48-year-old woman with a Klatskin tumor presented with obstructive jaundice. **a** Cholangiography shows a Bismuth type II hilar stricture. **b** Two 8 mm*6 cm self-expandable stents were successfully deployed across the stricture using the side-by-side technique. **c** Repeat cholangiography shows good passage of the contrast agent through the bilateral stents

The procedures were carried out under fluoroscopy with or without ultrasonic guidance. After successfully puncturing the right intrahepatic bile duct with a 22G Chiba needle (Cook, Bloomington, IN), a NEFF set was inserted into the bile duct. The outer cannula of the NEFF set was kept for cholangiography to evaluate the stricture site. Then, a 0.035-in. guidewire was advanced and the outer cannula of the NEFF set was exchanged with a 5F Headhunter or Cobra catheter (Cook, Bloomington, IN) to navigate through the obstruction. After passing through the stricture and subsequently measuring its length, a 6F or 8F sheath (Terumo, Tokyo, Japan) was inserted and the guidewire was kept inside with its tip in the distal duodenum. Then, the left bile duct was punctured and the same steps were then performed on the right side. Two bare SEMSs were advanced over the two guidewires on each side and deployed in the centers of the bilateral strictures. Each end of the stent should be 1.5 to 2 cm longer than the biliary stricture. The stents were placed across the sphincter of Oddi when the lower part of the common bile duct was involved. If the stricture was only at the hilum, the stents were merely placed intrabiliary to reduce the reflux of duodenal contents and decreases the risk of cholangitis. After stenting, repeat cholangiography was performed to verify stent patency and the two puncture paths were occluded through the sheaths using gelfoam pledgets (Fig. 1).

### SIS group

**Fig. 2:**
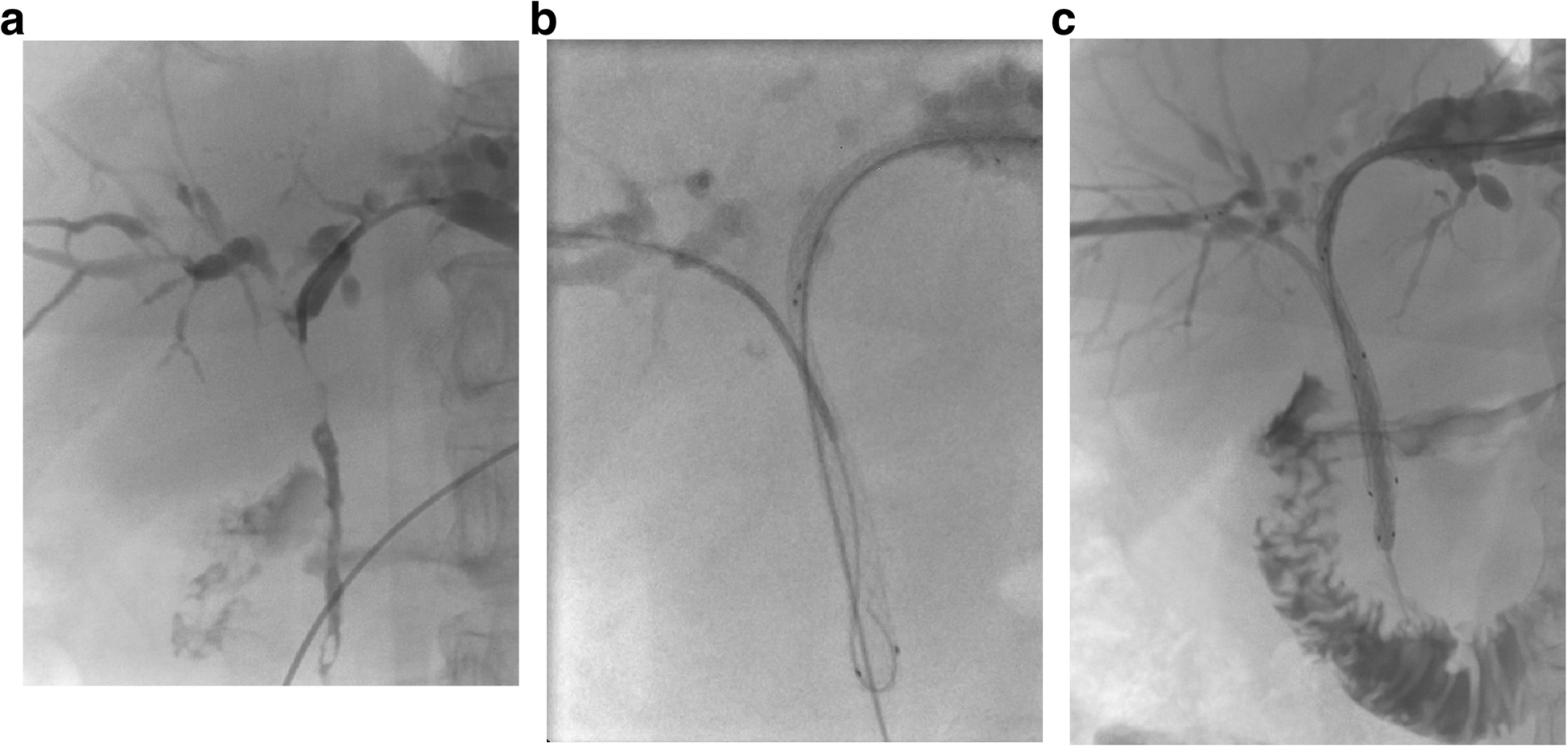
An 83-year-old woman had obstructive jaundice caused by gallbladder cancer. **a** A Bismuth type IIIa hilar stricture is demonstrated on cholangiography. **b** After the deployment of an 8 mm*6 cm stent from the left side, a 0.035-in. guidewire and a Headhunter catheter from the right side were inserted through the mesh of the stent to the duodenum. **c** Another 8 mm*6 cm stent was implanted from the right side with the stent-in-stent approach, and repeat cholangiography showed good patency of the two stents

The puncture technique was the same as that mentioned above. After successful stenting on one side, a 0.035-in. guidewire and a 5F Headhunter or Cobra catheter (Cook, Bloomington, IN) were inserted on the other side into the duodenum through the mesh of the contralateral stent at the biliary hilum. A 6F or 8F long sheath was inserted over the guidewire to dilate the mesh. Then, an uncovered SEMS was advanced and deployed across the stricture. Cholangiography was repeated to evaluate the bilateral stent patency and the two puncture paths were occluded with gelfoam pledgets (Fig. 2).

### Follow-up

After the procedure, the patients were regularly followed up to June 2019 or until the death of the patients. The follow-up visits consisted of outpatient and telephone interviews. Outpatient interviews were performed one month after stent placement. Telephone consultations were performed at two weeks and then every three months after stenting. If obstructive jaundice recurred which was confirmed by an elevated bilirubin level and dilated bile ducts on CT, the patient was encouraged to receive stent revision or external drainage.

### Technical and clinical assessment

Technical success was defined as successful deployment of the bilateral stents in the appropriate positions and good contrast flow through the stents into the duodenum.

The clinical outcome was evaluated in the following aspects, including the improvement of jaundice at 1 week and 1 month after the procedure, complications, stent patency period, and overall survival. A significant improvement of jaundice was defined as a decrease in serum bilirubin level of more than 20% at 1 week and more than 75% at 1 month after stent placement compared with the preoperative baseline [9, 10]. The complications were divided into major and minor complications according to the reporting standards of the Society of Interventional Radiology [11]. Major complications were defined as those requiring major therapy, an unplanned increase in level of care or prolonged hospitalization (> 48 h) or those causing permanent adverse sequelae or death. Other complications were regarded as minor. The stent patency period was defined as the time interval between the initial stent placement and recurrence of jaundice, the last follow-up or death of the patient without evidence of jaundice. If a patient died without recurrent jaundice, the stent patency period was considered to be the same as the duration of survival. Survival was calculated from the time of the initial stent placement to death from any cause or the last follow-up visit. Data on stent patency and survival were censored for patients who were alive at the time of writing this manuscript.

### Statistics

The independent t-test was used to compare continuous variables. The chi-squared test or Fisher’s exact test was used to compare categorical variables, depending on the scale level. Survival curves were calculated by the Kaplan–Meier method and compared by the log-rank test. A two-tailed *P*-value lower than 0.05 was considered statistically significant. All analyses were carried out using SPSS version 15.0 software (SPSS, Chicago, Illinois, USA).

## Results

Of the 65 patients included in this study, 37 were male and the other 28 were female, with a mean age of 64.1 years (range: 24–92). The primary causes of biliary obstruction were cholangiocarcinoma in 27 patients, gallbladder cancer in 17 patients, hepatocellular carcinoma in 11 patients, and metastatic nodes in 10 patients. According to the Bismuth classification of perihilar cholangiocarcinoma, 17 patients belonged to type II, 38 patients to type III, and 10 patients to type IV. The baseline characteristics of the patients in the two groups are listed in Table 1, and no significant differences were found between the two groups (*P* > 0.05).

**Table 1:** Patients’ characteristics of the two groups

Characteristics	SBS group	SIS group	*p* value
Pt. No.	38	27	
Gender			0.30
Male	19 (50.0)	10 (37.0)	
Female	19 (50.0)	17 (63.0)	
Age (y)	63.0 ± 12.4	65.3 ± 13.5	0.48
ECOG			0.48
0	3 (7.7)	2 (6.2)	
1	23 (61.5)	20 (75)	
2	12 (30.8)	5 (18.8)	
Obstruction causes			0.72
Cholangiocarcinoma	18 (47.3)	9 (33.4)	
Gallbladder cancer	9 (23.7)	8 (29.6)	
HCC	6 (15.8)	5 (18.5)	
Others	5 (13.2)	5 (18.5)	
Bismuth classification			0.68
Type II	9 (23.7)	8 (29.6)	
Type III	22 (57.9)	16 (59.3)	
Type IV	7 (18.4)	3 (11.1)	
Further chemotherapy	6 (15.8)	4 (14.8)	0.91

Note-Values presented as mean ± standard deviation where applicable. Values in parentheses are percentages

*ECOG* Eastern Cooperative Oncology Group, *HCC* Hepatocellular Carcinoma

Technical success was achieved in all patients. The most commonly used stents had sizes of 8*80 mm and 8*60 mm. One week after the procedures, a 20% reduction in serum bilirubin level was achieved in 89% (34/38) of the patients in the SBS group, and only in 67% (18/27) of the patients in the SIS group (*P* = 0.03). One month after stenting, however, there was no significant difference in the proportion of patients with a 75% reduction in serum bilirubin level between the two groups (SBS vs. SIS, 92% vs. 89%, P > 0.05).

Major complications only occurred in one patient in the SBS group. The patient was 79 years old and developed acute pancreatitis after the procedure. Antibiotics and protein degeneration enzyme inhibitors were given. However, the patient’s condition did not improve, and he died of renal failure eight days after the procedure. Minor complications, including cholangitis, cholecystitis, pancreatitis, and peritonitis, occurred in 18% (7/38) of the patients in the SBS group and 33% (9/27) of the patients in the SIS group (*P* > 0.05). Cholangitis was found to be more frequent among the patients in the SIS group than among those in the SBS group (30% vs. 8%, *P* = 0.04).

**Fig. 3:**
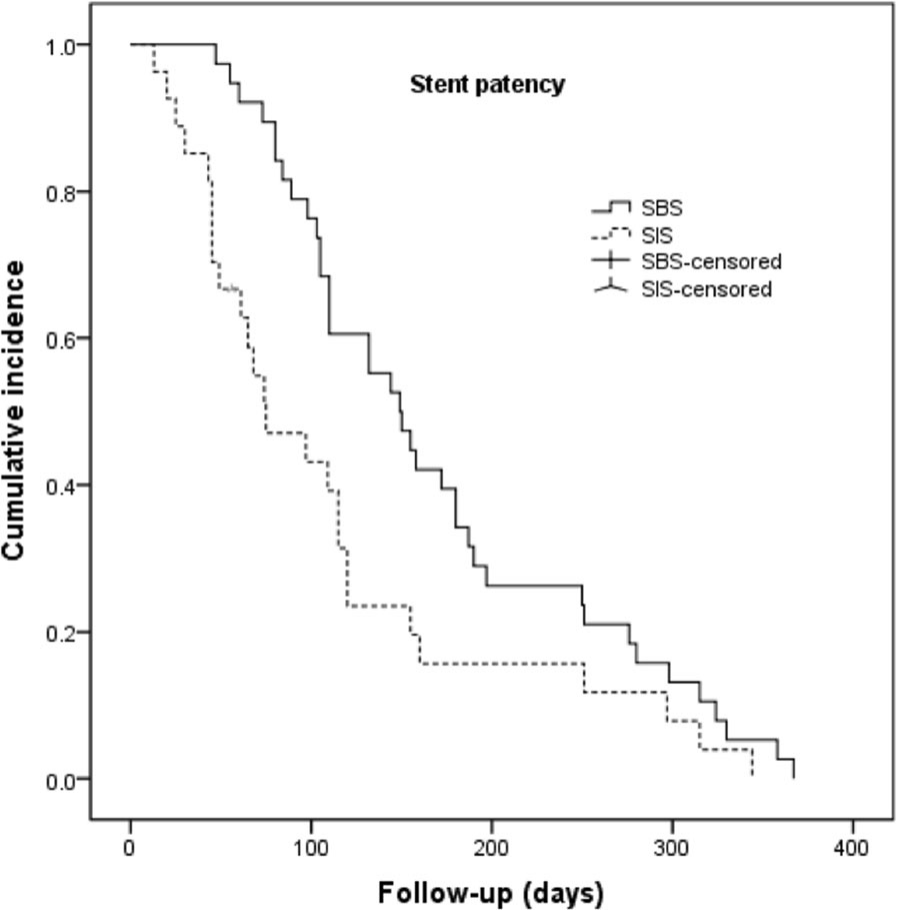
Kaplan-Meier estimation of stent patency. The stent patency period was significantly longer in the SBS group than in the SIS group (*P* = 0.02)

**Fig. 4:**
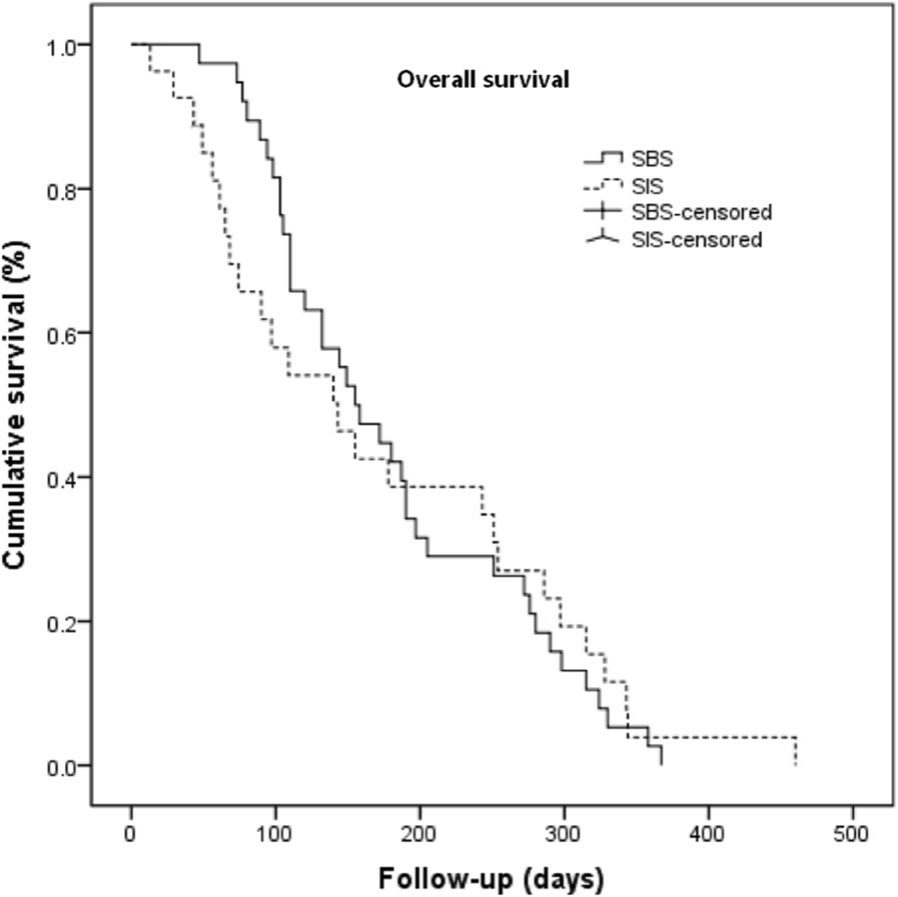
Kaplan-Meier estimation of patient survival. No significant difference was found between the two groups (*P* > 0.05)

The median follow-up time was 6 months (range, 2–12 months) in the SBS group and 5 (range, 2–14 months) months in the SIS group. The median stent patency duration was significantly longer in the SBS group (149 days) than in the SIS group (75 days; *P* = 0.02). (Fig. 3). During the follow-up period, stent occlusion occurred in seven patients in the SBS group (stent duration: 55–250 days) and 10 patients in the SIS group (stent duration: 20–160 days) (*P* > 0.05). Among these patients, nine patients underwent external drainage, seven patients underwent stent revision using new stents, and one patient only received conservative therapies due to poor general conditions. The median overall survival in the SBS group was 155 days, which did not differ significantly from the 143 days in the SIS group (P > 0.05) (Fig. 4). The main results of the two groups are listed in Table 2.

**Table 2:** Main results of the two groups

Outcomes	SBS group (*n* = 38)	SIS group (*n* = 27)	p value
Technical success	38 (100)	27 (100)	1
Reduction of bilirubin level			
By 20% at 1 week	34 (89.5)	18 (66.7)	0.03
By 75% at 1 month	35 (92.1)	24 (88.9)	0.69
Complications			
Major complication			1
Acute renal failure	1 (2.6)	0	
Minor complications	7 (18.4)	9 (33.3)	0.24
Cholangitis	3 (7.9)	8 (29.6)	0.04
Cholecystitis	1 (2.6)	0	1
Pancreatitis	2 (5.3)	1 (3.7)	1
Peritonitis	1 (2.6)	0	1
Stent occlusion	7 (18.4)	10 (37)	0.09

Note-Values in parentheses are percentages

## Discussion

In patients with malignant hilar biliary strictures, drainage of more than 50% of the liver volume usually requires bilateral stent implantation unless there is a definite hypertrophic lobe [12]. Several retrospective studies have shown that bilateral metal stent insertion leads to longer stent patency and patient survival than unilateral metal or plastic stent insertion [13–15]. Bilateral stent placement can be performed endoscopically or percutaneously [16, 17]. A large series study revealed that transpapillary stent placement can facilitate endoscopic retreatment, which has a positive impact on the patient’s quality of life [16]. However, in most situations, the decision to use either an endoscopic or percutaneous approach mainly depends on local expertise and patient’s cost [18].

Currently, two methods of stent placement are most commonly used for hilar strictures: SBS and SIS. Previously, there were only a few studies that compared these two different techniques. In 2012, Naitoh et al. [5] initially evaluated these two techniques and found that the SBS technique tended to lead to a longer stent patency than the SIS technique, although the SBS technique had more complications. However, later studies did not find any significant differences in technical success, complications, or stent occlusion [6–8]. Thus, it is still contrroversial which technique is better. Additionally, all the stents in these studies were placed under endoscopy, and therehas been no comparison of these two techniques performed via the percutaneous transhepatic approach.

In this study, the stents were all placed percutaneously using these two techniques. The technical success rate was 100% in both groups. However, in the SIS group, the mesh of the first stent must be passed through before deploying the second stent, usually requiring a long sheath or a balloon catheter to dilate the mesh. Thus, the SIS approach is more time consuming and technically demanding. In addition, we found that the bilirubin level after the procedures was reduced more quickly in the SBS group than in the SIS group. This may be due to the different stent configurations in the two groups. The two parallel stents in the SBS group offer a larger flow area in the common hepatic duct than the two stents with an SIS configuration, which may allow the bile to be drained more effectively.

In addition, the obstructive jaundice improved quicker in the SBS group, and the duration of stent patency was also longer in the SBS group than that in the SIS group. Theoretically, the SBS configuration provides a larger flow area than the SIS configuration, which may delay the event of a stent obstruction in the common bile duct caused by tumor ingrowth.

In this study, the procedure-related complication rates were 18.4% in the SBS group and 33.3% in the SIS group, which were similar to the rates in the papers that performed the procedures endoscopically [5, 6]. Noticeably, cholangitis was less frequently observed in the SBS group than in the SIS group. Several studies have demonstrated that incomplete biliary drainage is a significant risk factor for postoperative cholangitis [19–21]. In patients with Bismuth III and IV strictures, more than two stents are often required to drain all opacified ducts [16]. In our study, these patients only received two stents, which could cause subsequent cholangitis due to undrained ducts. However, the composition of the two groups were similar, which could offset this impact. Thus, the bile may be drained more effectively in the SBS group owing to the large flow area of the common hepatic duct, which could decrease the rate of bile infection. This result is different from a previous study [5]. It has been reported that excessive expansion of the two SBS stents might cause portal vein occlusion and increase the rate of cholangitis [5].

This study has several limitations. First, this was a retrospective study with a small number of patients. Second, the selection of stent type and the technique of stent deployment mainly relied on the operators’ preferences in this study, which may introduce bias and influence the outcome. Therefore, a prospective, randomized controlled trial with the same type of stent is warranted to further clarify the differences between the SBS and SIS techniques performed through the percutaneous approach.

## Conclusions

In conclusion, percutaneous transhepatic bilateral stenting using either the SBS or SIS techniques is safe and effective for the treatment of malignant hilar biliary obstruction. Compared with SIS, SBS offers quicker improvement of jaundice, a lower incidence of cholangitis after the procedure, and a longer stent patency period.

## Data Availability

The datasets used and analysed during the current study are available from the corresponding author on reasonable request.

## Abbreviations

SBS: Side-by-side
SIS: Stent-in-stent
ECOG: Eastern Cooperative Oncology Group
HCC: Hepatocellular Carcinoma

## References

[1] Deipolyi AR, Covey AM (2017). Palliative percutaneous biliary interventions in malignant high bile duct obstruction. Semin Intervent Radiol.

[2] Nam HS, Kang DH (2016). Current status of biliary metal stents. Clin Endosc.

[3] Dumonceau JM, Heresbach D, Deviere J, Costamagna G, Beilenhoff U, Riphaus A (2011). Biliary stents: models and methods for endoscopic stenting. Endoscopy.

[4] Hong W, Chen S, Zhu Q, Chen H, Pan J, Huang Q (2014). Bilateral stenting methods for hilar biliary obstructions. Clinics (Sao Paulo).

[5] Naitoh I, Hayashi K, Nakazawa T (2012). Side-by-side versus stent-in-stent deployment in bilateral endoscopic metal stenting for malignant hilar biliary obstruction. Dig Dis Sci.

[6] Kim KM, Lee KH, Chung YH (2012). A comparison of bilateral stenting methods for malignant hilar biliary obstruction. Hepatogastroenterology.

[7] Law R, Baron TH (2013). Bilateral metal stents for hilar biliary obstruction using a 6Fr delivery system: outcomes following bilateral and side-by-side stent deployment. Dig Dis Sci.

[8] Lee TH, Moon JH, Choi JH (2019). Prospective comparison of endoscopic bilateral stent-in-stent versus stent-by-stent deployment for inoperable advanced malignant hilar biliary stricture. Gastrointest Endosc.

[9] Brountzos EN, Ptochis N, Panagiotou I, Malagari K, Tzavara C, Kelekis D (2007). A survival analysis of patients with malignant biliary strictures treated by percutaneous metallic stenting. Cardiovasc Intervent Radiol.

[10] Gwon DI, Ko GY, Kim JH (2013). Percutaneous bilateral metallic stent placement using a stentin-stent deployment technique in patients with malignant hilar biliary obstruction. AJR Am J Roentgenol.

[11] Sacks D, McClenny TE, Cardella JF, Lewis CA (2003). Society of Interventional Radiology clinical practice guidelines. J Vasc Interv Radiol.

[12] Vienne A, Hobeika E, Gouya H (2010). Prediction of drainage effectiveness during endoscopic stenting of malignant hilar strictures: the role of liver volume assessment. Gastrointest Endosc.

[13] Chang WH, Kortan P, Haber GB (1998). Outcome in patients with bifurcation tumors who undergo unilateral versus bilateral hepatic duct drainage. Gastrointest Endosc.

[14] Naitoh I, Ohara H, Nakazawa T (2009). Unilateral versus bilateral endoscopic metal stenting for malignant hilar biliary obstruction. J Gastroenterol Hepatol.

[15] Liberato MJ, Canena JM (2012). Endoscopic stenting for hilar cholangiocarcinoma: efficacy of unilateral and bilateral placement of plastic and metal stents in a retrospective review of 480 patients. BMC Gastroenterol.

[16] Boškoski I, Tringali A, Familiari P (2019). A 17 years retrospective study on multiple metal stents for complex malignant hilar biliary strictures: survival, stents patency and outcomes of re-interventions for occluded metal stents. Dig Liver Dis.

[17] Paik WH, Park YS, Hwang JH (2009). Palliative treatment with self-expandable metallic stents in patients with advanced type III or IV hilar cholangiocarcinoma: a percutaneous versus endoscopic approach. Gastrointest Endosc.

[18] Dumonceau JM, Tringali A, Papanikolaou IS (2018). Endoscopic biliary stenting: indications, choice of stents, and results: European Society of Gastrointestinal Endoscopy (ESGE) clinical guideline – updated October 2017. Endoscopy..

[19] Voiosu TA, Bengus A, Haidar A (2014). Antibiotic prophylaxis prior to elective ERCP does not alter cholangitis rates or shorten hospital stay: results of an observational prospective study of 138 consecutive ERCPs. Maedica (Buchar).

[20] Ishigaki T, Sasaki T, Serikawa M (2015). Evaluation of antibiotic use to prevent post-endoscopic retrograde cholangiopancreatography pancreatitis and cholangitis. Hepatogastroenterology.

[21] Wobser H, Gunesch A, Klebl F (2017). Prophylaxis of post-ERC infectious complications in patients with biliary obstruction by adding antimicrobial agents into ERC contrast media – a single center retrospective study. BMC Gastroenterol.

